# Our Bureau of Information

**Published:** 1911-10-28

**Authors:** 


### OUR BUREAU OF INFORMATION.

### Practical Help for all Workers among the Sick.

In this week's issue will be found, at the bottom of the third inner page of the cover, a special coupon. The coupon is the practical intimation of a new departure which we have decided to make in order to try and help workers among the sick and all classes of dependants to discharge their often difficult duties with the maximum of speed and success. Many members of the medical profession, many clergymen, many superintendents, matrons, sisters, nurses, visitors, and workers of all kinds are often disturbed in mind for the want of the means to put them in the way of promptly dealing with a difficult case which may require special care or other help of a practical kind. They frequently do not know where to turn for the information they require, or how to procure the means without which the unfortunate object of their solicitude must suffer avoidable trials, and may even be deprived of the opportunity to contribute towards their own maintenance and comfort.

The means are usually in existence. In these cases the difficulty arises from a want of intimate knowledge of all the agencies of every kind which are available to meet almost every kind of human need and disablement. For upwards of forty years it has been our privilege to collect information, to study closely the actual provision which is available, and to make ourselves familiar with the work and objects and means of institutions of all kinds. Every day inquiries reach us from all kinds of people and even from many parts of the world, and we have endeavoured to the best of our ability to co-operate with those who find themselves in a difficulty and to help them out of it.

The time seems therefore to have come readily to make available this bureau of information, to which every worker may have free access through our columns by becoming a reader of The Hospital. If it is to prove practically useful we shall require the co-operation of our readers not only by making application to the Editor for help of all kinds when they need it, but by examining the cases and inquiries which we shall publish from week to week and sending whenever possible suggestions or offers of help in regard to particular cases mentioned on this page. It would be a task of human impossibility for any editor to cover anything like the wide field which is here presented for cultivation, unless he had behind him the co-operation and sympathy of every institutional worker who makes The Hospital a companion and friend.

Here then is presented a means for doing a great amount of personal service to those unfortunates who, from no fault of their own, find themselves in a most difficult and often cruel position, when their power of self-maintenance or work is diminished or destroyed by circumstances beyond their control. These are the sad cases which we are certain will call forth and secure full co-operation at all times from our readers. Let us then join hands in an earnest endeavour to make this page as practically useful as possible.

### How to make the Bureau a Success.

In order to facilitate as far as possible the work we have in view, everyone who uses or wishes to help our Bureau of Information must be kind enough to observe the following rules:—

(1) Postal replies cannot be given. Exception will only be made in very special cases at the Editor's discretion.

(2) Queries or answers relating to different cases must be written, or preferably typed, on separate sheets of paper, and the writing must never be crossed.

(3) Questions and replies received not later than Wednesday will, as far as possible, be dealt with in our issue of the following Saturday week.

(4) Letters must have attached the name and address of the sender, with a pseudonym for publication if preferred.

(5) *All communications* for this department *must be accompanied by the coupon* to be found at the bottom of the third page of the cover on the inside, and should be addressed to the Editor of the Bureau of Information, c/o The Hospital, 29 Southampton Street, Strand, London, W.C.

*Notice.*—We invite workers of special experience and knowledge to offer help in replying to questions relating to their special department.

Remember the object of the Bureau is to be practically helpful directly to workers amongst the sick, and indirectly to every sufferer who needs information and help. Knowledge saves time and shortens suffering. It helps the unfortunate, strengthens the worker, and tends to make us fuller men and women. We cannot all be Solomons, but by co-operation and intercommunication, being workers in the same field and imbued with the same desire to make this a brighter and happier world, if possible, together we can do many things which would be otherwise beyond our powers of accomplishment. We have collected much practical information, but, like truth, it must lie buried unless our readers wake up and test its value and make this Bureau as widely known as possible. Let none of us forget what Shakespeare has well said: "Ignorance is the curse of God, knowledge the wing with which we fly to Heaven."

### Needs and Helpers.

### INSTITUTION FOR INCURABLE CONSUMPTION.

"Kent" writes: I am urgently in need of information as to the form of application and conditions of admission to such an institution for one such sufferer, a woman. I want to have the addresses of one or more of these institutions, and I need all the help you can give to secure for her the best surroundings to cheer and help her to the end.

[No mention is made of the social condition of the patient referred to, but it would appear as if some payment may be possible. We should be glad to have suggestions, information, and an intimation of any vacancy which may fulfil the requirements of this case.—Ed. The Hospital.]

